# *N*-[(9*H*-Fluoren-9-yl­idene)(2-meth­oxy­phen­yl)meth­yl]-1,1,1-tri­methyl­silanamine

**DOI:** 10.1107/S1600536813033424

**Published:** 2013-12-14

**Authors:** Zhong-Yuan Li, Peng Wang, Xia Chen

**Affiliations:** a School of Chemistry and Chemical Engineering, Shanxi University, Taiyuan 030006, People’s Republic of China

## Abstract

The title mol­ecule, C_24_H_25_NOSi, is a hydrolysis product of the reaction between 9-tri­methyl­silyfluorenyl lithium and 2-meth­oxy­benzo­nitrile. The fluorene ring system is substanti­ally planar, with an r.m.s. deviation of 0.0288 Å from the best-fit plane through its 13 C atoms. This plane forms a dihedral angle of 58.07 (7)° with the 2-meth­oxy­benzyl­amine ring plane. In the crystal, mol­ecules are linked by N—H⋯π and C—H⋯π inter­actions, which leads to the formation of two-dimensional network lying parallel to the *bc* plane.

## Related literature   

For the use of fluorene as a ligand in organometallic chemistry, see: Alt & Samuel (1998 ▶); Kirillov *et al.* (2005 ▶); Bochmann *et al.* (1993 ▶); Decken *et al.* (2002 ▶); Knjazhanski *et al.* (2002 ▶); Novikova *et al.* (1985 ▶); Johnson & Treichel (1977 ▶). For σ–π stacking, see: Calhorda (2000 ▶); Desiraju & Steiner (1999 ▶). For a related amino­fulvene structure, see: Axenov *et al.* (2009 ▶).
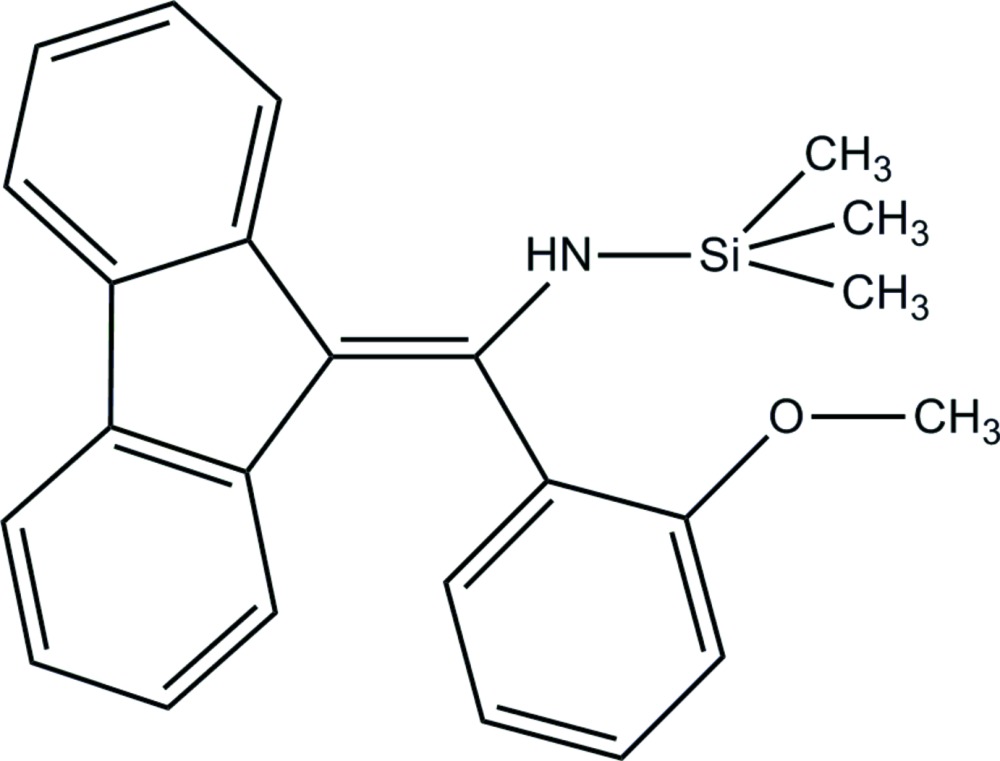


## Experimental   

### 

#### Crystal data  

C_24_H_25_NOSi
*M* *_r_* = 371.54
Monoclinic, 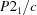

*a* = 12.611 (3) Å
*b* = 9.5694 (19) Å
*c* = 20.325 (6) Å
β = 124.10 (2)°
*V* = 2031.1 (9) Å^3^
*Z* = 4
Mo *K*α radiation
μ = 0.13 mm^−1^
*T* = 173 K
0.19 × 0.17 × 0.12 mm

#### Data collection  

Bruker P4 diffractometer
Absorption correction: multi-scan (*SADABS*; Sheldrick, 1996 ▶) *T*_min_ = 0.976, *T*_max_ = 0.985
15991 measured reflections
4628 independent reflections
4212 reflections with *I* > 2σ(*I*)
*R*_int_ = 0.057

#### Refinement  

*R*[*F* ^2^ > 2σ(*F* ^2^)] = 0.075
*wR*(*F* ^2^) = 0.173
*S* = 1.24
4628 reflections
248 parameters
H-atom parameters constrained
Δρ_max_ = 0.50 e Å^−3^
Δρ_min_ = −0.26 e Å^−3^

### 

Data collection: *SMART* (Bruker, 2000 ▶); cell refinement: *SAINT* (Bruker, 2000 ▶); data reduction: *SAINT*; program(s) used to solve structure: *SHELXS97* (Sheldrick, 2008 ▶); program(s) used to refine structure: *SHELXL97* (Sheldrick, 2008 ▶); molecular graphics: *SHELXTL/PC* (Sheldrick, 2008 ▶); software used to prepare material for publication: *SHELXL97*.

## Supplementary Material

Crystal structure: contains datablock(s) I. DOI: 10.1107/S1600536813033424/sj5378sup1.cif

Structure factors: contains datablock(s) I. DOI: 10.1107/S1600536813033424/sj5378Isup2.hkl

Supporting information file. DOI: 10.1107/S1600536813033424/sj5378Isup3.cml

Additional supporting information:  crystallographic information; 3D view; checkCIF report

## Figures and Tables

**Table 1:** Hydrogen-bond geometry (Å, °) *Cg*1, *Cg*2 and *Cg*4 are the centroids of the C1,C2,C7,C8,C13, C2–C7 and C15–C20 rings, respectively.

*D*—H⋯*A*	*D*—H	H⋯*A*	*D*⋯*A*	*D*—H⋯*A*
N1—H1⋯*Cg*1^i^	0.88	2.69	3.347 (3)	133
C12—H12*A*⋯*Cg*4	0.95	2.99	3.750 (4)	138
C16—H16*A*⋯*Cg*2^i^	0.95	2.65	3.470 (3)	145
C21—H21*C*⋯*Cg*2^ii^	0.98	2.94	3.736 (4)	139
C24—H24*A*⋯*Cg*3^i^	0.98	2.99	3.923 (4)	158

Symmetry codes: (i) 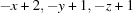; (ii) 
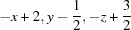
.

**Fig. 1.**
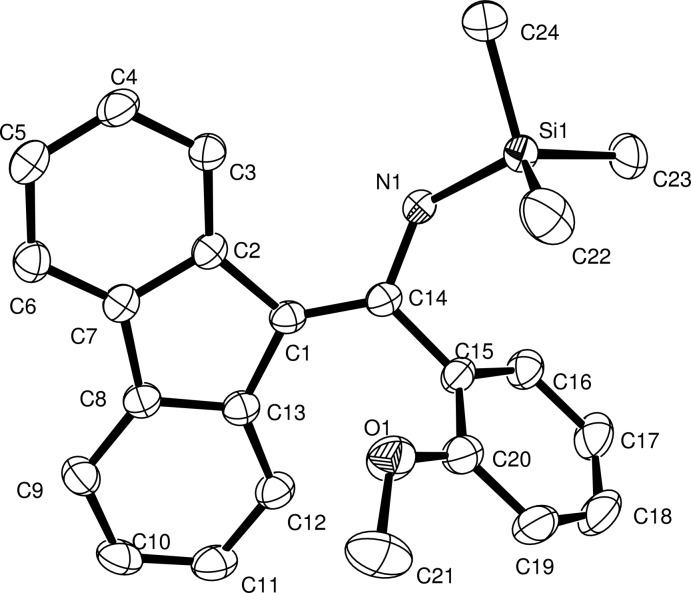
The molecular structure, showing the atom–numbering scheme. Displacement ellipsoids are drawn at the 30% probability level. Hydrogen atoms are omitted for clarity.

**Fig. 2.**
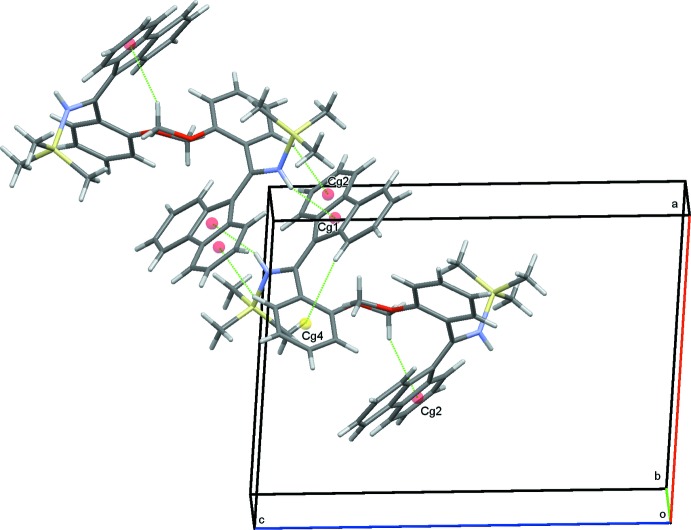
Crystal packing of 1 with N–H···π and C–H···π contacts drawn as dotted lines and spheres representing the aromatic ring centroids.

## supplementary crystallographic information

### 1.1. Synthesis and crystallization

The 9-tri­methyl­silyl-fluorenyllithium (0.68 g, 2.8 mmol) mixed with (o-MeO)PhCN (0.34 ml, 2.8 mmol) at 0 °C. The resulting mixture was slowly warmed to room temperature and stirred for a further 10 hours to give a clear brown solution. H_2_O (2.8 mmol, 0.41 ml, 6.94 *M* in THF) was added to a stirred solution, prepared *in situ* without purification, at 0 °C. The resulting cloudy yellow solution was allowed to warm to room temperature for 7 days, yielding colorless crystals of the title compound (0.62 g, 59% yield). Mp: 172 °C. ^1^H NMR (300 MHz, C_6_D_6_): *δ* (ppm) -0.14 (s, 9H, -Si(CH_3_)_3_), 2.68 (s,1H,-NH), 3.78 (s, 3H, -OCH_3_), 6.71 (s, 2H, -CH- of phenyl), 6.88-6.90 (d, *J*_HH_=7.5 Hz, 2H, -CH- of fluorenyl), 7.21-7.44 (m, 2H, -CH- of phenyl), 7.59-7.63 (m, 2H, -CH- of fluorenyl), 7.71-7.73 (d, *J*_HH_=7.8 Hz, 2H, -CH- of fluorenyl), 7.89-7.92 (d, *J*_HH_=7.8 Hz, 2H, -CH- of fluorenyl). ^13^C NMR (75 MHz, CDCl_3_): *δ* (ppm) 1.99 (3C, C of -SiMe_3_), 58.23 (1C, -OCH3), 109.33, 113.23, 120.27, 121.02, 122.11, 124.21, 126.35, 127.33, 128.53, 138.40, 139.24, 140.12, 140.35, 141.11, 143.23 (17C, C of fluorenyl and phenyl), 151.78, 166.65 (2C, Cipso of phenyl), 154.32 (1C, PhCNHSiMe_3_). Anal. Calc. for C_24_H_25_NOSi (Mr = 371.55): C, 77.58; H, 6.78; N, 3.77%. Found: C, 77.80; H, 6.68; N, 3.82%.

### 1.2. Refinement

The methyl H atoms were constrained to an ideal geometry, with C—H distances of 0.98 Å and *U*_iso_(H) = 1.5*U*_eq_(C). N–H bond distances was restrained to be 0.88 Å and *U*_iso_(H) = 1.2*U*_eq_(N). The phenyl H atoms were placed in geometrically idealized positions and constrained to ride on their parent atoms, with C—H distances of 0.95 Å and *U*_iso_(H) = 1.2*U*_eq_(C).

### 2. Comment

Fluorene is an attractive ligand for organometallic chemistry for several reasons. It may be regarded as a doubly benzannelated cyclo­penta­diene, which may be deprotonated at the 9 position to generate a substituted Cp ligand. Indeed, it is this unit upon which much of the organometallic chemistry of fluorene is based. This ligand may bind to metals in a wide variety of ways, many of which are unavailable to analogous Cp species, with η^1^, η^3^, and η^5^ forms all structurally characterized (Alt and Samuel, 1998; Kirillov *et al.*, 2005; Bochmann *et al.*, 1993; Decken *et al.*, 2002; Knjazhanski *et al.*, 2002). Fluorene may also be regarded as a CH_2_-bridged bi­phenyl unit, with two potential binding sites on the arene rings. Again, this has been exploited, with the synthesis of several bimetallic systems with the ligand again showing the ability to bind in a variety of coordination modes, η^5^ and η^6^ are both known (Novikova *et al.*, 1985; Johnson and Treichel, 1977). Here, we report the synthesis and structure of the new compound N-((9H-fluoren-9-yl­idene)(2-meth­oxy­phenyl)­methyl)-1,1,1-tri­methyl­silanamine.

The molecular structure of the title compound is illustrated in Fig. 1. The compound is a hydrolysis product of the reaction between 9-tri­methyl­silyfluorenyl lithium and 2-meth­oxy­benzo­nitrile. The fluorene ring system is substanti­ally planar with an rms deviation of 0.0288 Å from the best fit plane through its 13 C atoms. This plane forms a dihedral angle of 58.07 (7)° with the 2-meth­oxy­benzo­nitrile ring plane. The five-membered shows alternating C=C and C—C bond length. The exocyclic C1—C14 [1.368 (4)Å] linkage is in the typical double bond range [1.32 Å]. This comound contains a typical amino­fulvene framework (Axenov *et al.*, 2009). The adjacent C14—N1 bond is also short, indicating the presence of delocalization in the C1—C14—N1 fragments to some extent. The other adjacent bond distance, C14—C15, is 1.490 (3)Å which is in agreement with single bond character [1.53 Å]. A number of N–H···π and C–H···π stacking inter­actions involving the phenyl rings help to consolidate the crystal packing. The N···Cg and C···Cg (Cg = ring centroid) distances lie in the range 2.989-3.473 Å, which is normal for such inter­actions (Calhorda, 2000; Desiraju & Steiner, 1999) and lead to the formation of an infinite one-dimensional chain structure (Fig. 2).

### Crystal data

**Table:**

C_24_H_25_NOSi	*F*(000) = 792
*M**_r_* = 371.54	*D*_x_ = 1.215 Mg m^−^^3^
Monoclinic, *P*2_1_/*c*	Mo *K*α radiation, λ = 0.71073 Å
Hall symbol: -P 2ybc	Cell parameters from 6083 reflections
*a* = 12.611 (3) Å	θ = 1.6–27.5°
*b* = 9.5694 (19) Å	µ = 0.13 mm^−^^1^
*c* = 20.325 (6) Å	*T* = 173 K
β = 124.10 (2)°	Prism, yellow
*V* = 2031.1 (9) Å^3^	0.19 × 0.17 × 0.12 mm
*Z* = 4	

### Data collection

**Table:**

Bruker P4 diffractometer	4628 independent reflections
Radiation source: fine-focus sealed tube	4212 reflections with *I* > 2σ(*I*)
Graphite monochromator	*R*_int_ = 0.057
ω scans	θ_max_ = 27.4°, θ_min_ = 2.7°
Absorption correction: multi-scan (*SADABS*; Sheldrick, 1996)	*h* = −16→12
*T*_min_ = 0.976, *T*_max_ = 0.985	*k* = −12→12
15991 measured reflections	*l* = −25→26

### Refinement

**Table:**

Refinement on *F*^2^	Primary atom site location: structure-invariant direct methods
Least-squares matrix: full	Secondary atom site location: difference Fourier map
*R*[*F*^2^ > 2σ(*F*^2^)] = 0.075	Hydrogen site location: inferred from neighbouring sites
*wR*(*F*^2^) = 0.173	H-atom parameters constrained
*S* = 1.24	*w* = 1/[σ^2^(*F*_o_^2^) + (0.0619*P*)^2^ + 1.2002*P*] where *P* = (*F*_o_^2^ + 2*F*_c_^2^)/3
4628 reflections	(Δ/σ)_max_ < 0.001
248 parameters	Δρ_max_ = 0.50 e Å^−^^3^
0 restraints	Δρ_min_ = −0.26 e Å^−^^3^

### Special details

**Table:**

Geometry. All e.s.d.'s (except the e.s.d. in the dihedral angle between two l.s. planes) are estimated using the full covariance matrix. The cell e.s.d.'s are taken into account individually in the estimation of e.s.d.'s in distances, angles and torsion angles; correlations between e.s.d.'s in cell parameters are only used when they are defined by crystal symmetry. An approximate (isotropic) treatment of cell e.s.d.'s is used for estimating e.s.d.'s involving l.s. planes.
Refinement. Refinement of *F*^2^ against ALL reflections. The weighted *R*-factor *wR* and goodness of fit *S* are based on *F*^2^, conventional *R*-factors *R* are based on *F*, with *F* set to zero for negative *F*^2^. The threshold expression of *F*^2^ > σ(*F*^2^) is used only for calculating *R*-factors(gt) *etc*. and is not relevant to the choice of reflections for refinement. *R*-factors based on *F*^2^ are statistically about twice as large as those based on *F*, and *R*- factors based on ALL data will be even larger.

### Fractional atomic coordinates and isotropic or equivalent isotropic displacement parameters (Å^2^)

**Table:**

	*x*	*y*	*z*	*U*_iso_*/*U*_eq_	
Si1	0.66989 (6)	0.64695 (7)	0.46564 (4)	0.02799 (19)	
O1	0.94557 (19)	0.4521 (2)	0.70940 (11)	0.0421 (5)	
N1	0.81941 (18)	0.5781 (2)	0.49531 (11)	0.0270 (4)	
H1	0.8454	0.6009	0.4646	0.032*	
C1	1.0326 (2)	0.5139 (2)	0.60208 (13)	0.0253 (5)	
C2	1.0993 (2)	0.6446 (2)	0.60736 (14)	0.0253 (5)	
C3	1.0568 (2)	0.7744 (2)	0.56909 (15)	0.0300 (5)	
H3A	0.9696	0.7869	0.5268	0.036*	
C4	1.1426 (2)	0.8843 (3)	0.59324 (16)	0.0334 (6)	
H4A	1.1131	0.9719	0.5670	0.040*	
C5	1.2708 (3)	0.8693 (3)	0.65494 (16)	0.0353 (6)	
H5A	1.3276	0.9464	0.6708	0.042*	
C6	1.3158 (2)	0.7417 (3)	0.69335 (15)	0.0315 (5)	
H6A	1.4034	0.7305	0.7354	0.038*	
C7	1.2307 (2)	0.6306 (2)	0.66934 (14)	0.0268 (5)	
C8	1.2521 (2)	0.4893 (2)	0.70131 (14)	0.0274 (5)	
C9	1.3646 (2)	0.4240 (3)	0.76098 (15)	0.0342 (6)	
H9A	1.4436	0.4732	0.7886	0.041*	
C10	1.3589 (3)	0.2860 (3)	0.77916 (16)	0.0388 (6)	
H10A	1.4346	0.2403	0.8199	0.047*	
C11	1.2439 (3)	0.2140 (3)	0.73847 (16)	0.0386 (6)	
H11A	1.2421	0.1191	0.7515	0.046*	
C12	1.1311 (2)	0.2778 (3)	0.67909 (16)	0.0332 (6)	
H12A	1.0532	0.2265	0.6511	0.040*	
C13	1.1335 (2)	0.4186 (2)	0.66094 (14)	0.0271 (5)	
C14	0.9032 (2)	0.4938 (2)	0.55932 (14)	0.0257 (5)	
C15	0.8397 (2)	0.3807 (2)	0.57581 (15)	0.0278 (5)	
C16	0.7537 (2)	0.2919 (3)	0.51407 (16)	0.0347 (6)	
H16A	0.7369	0.3050	0.4627	0.042*	
C17	0.6921 (3)	0.1846 (3)	0.5262 (2)	0.0446 (7)	
H17A	0.6342	0.1243	0.4838	0.054*	
C18	0.7167 (3)	0.1672 (3)	0.6009 (2)	0.0517 (8)	
H18A	0.6748	0.0943	0.6096	0.062*	
C19	0.8008 (3)	0.2535 (3)	0.66320 (19)	0.0452 (7)	
H19A	0.8163	0.2395	0.7142	0.054*	
C20	0.8628 (2)	0.3610 (3)	0.65138 (16)	0.0340 (6)	
C21	0.9910 (3)	0.4175 (4)	0.78948 (17)	0.0574 (9)	
H21A	1.0579	0.4837	0.8258	0.086*	
H21B	1.0262	0.3226	0.8014	0.086*	
H21C	0.9199	0.4225	0.7962	0.086*	
C22	0.6655 (3)	0.6740 (4)	0.55400 (18)	0.0524 (8)	
H22A	0.6696	0.5834	0.5778	0.079*	
H22B	0.5857	0.7216	0.5383	0.079*	
H22C	0.7387	0.7314	0.5928	0.079*	
C23	0.5336 (3)	0.5378 (3)	0.39032 (18)	0.0457 (7)	
H23A	0.5327	0.4500	0.4148	0.069*	
H23B	0.5430	0.5176	0.3466	0.069*	
H23C	0.4532	0.5882	0.3698	0.069*	
C24	0.6621 (3)	0.8163 (3)	0.41832 (18)	0.0424 (7)	
H24A	0.6755	0.8001	0.3758	0.064*	
H24B	0.7289	0.8789	0.4582	0.064*	
H24C	0.5779	0.8590	0.3959	0.064*	

### Atomic displacement parameters (Å^2^)

**Table:**

	*U*^11^	*U*^22^	*U*^33^	*U*^12^	*U*^13^	*U*^23^
Si1	0.0246 (3)	0.0260 (3)	0.0320 (4)	−0.0010 (3)	0.0151 (3)	0.0018 (3)
O1	0.0480 (11)	0.0507 (12)	0.0328 (10)	−0.0042 (9)	0.0258 (9)	0.0010 (9)
N1	0.0255 (10)	0.0295 (10)	0.0279 (10)	−0.0011 (8)	0.0162 (9)	0.0041 (8)
C1	0.0286 (11)	0.0223 (11)	0.0279 (11)	0.0025 (9)	0.0177 (10)	0.0025 (9)
C2	0.0277 (11)	0.0242 (11)	0.0284 (12)	−0.0011 (9)	0.0184 (10)	−0.0016 (9)
C3	0.0268 (12)	0.0259 (12)	0.0363 (13)	0.0012 (9)	0.0171 (11)	0.0031 (10)
C4	0.0379 (13)	0.0217 (11)	0.0429 (15)	−0.0013 (10)	0.0240 (12)	0.0028 (10)
C5	0.0379 (14)	0.0274 (13)	0.0419 (15)	−0.0089 (11)	0.0232 (12)	−0.0042 (11)
C6	0.0297 (12)	0.0320 (13)	0.0303 (13)	−0.0047 (10)	0.0154 (11)	−0.0037 (10)
C7	0.0289 (12)	0.0267 (12)	0.0283 (12)	−0.0017 (9)	0.0182 (10)	−0.0020 (9)
C8	0.0302 (12)	0.0273 (12)	0.0270 (12)	0.0003 (9)	0.0174 (10)	−0.0001 (9)
C9	0.0274 (12)	0.0404 (14)	0.0310 (13)	0.0025 (10)	0.0140 (11)	0.0035 (11)
C10	0.0370 (14)	0.0384 (15)	0.0372 (14)	0.0144 (12)	0.0184 (12)	0.0127 (12)
C11	0.0459 (15)	0.0293 (13)	0.0458 (16)	0.0080 (12)	0.0288 (13)	0.0109 (12)
C12	0.0353 (13)	0.0268 (12)	0.0409 (14)	0.0012 (10)	0.0235 (12)	0.0036 (11)
C13	0.0302 (12)	0.0265 (12)	0.0282 (12)	0.0013 (9)	0.0186 (10)	0.0009 (9)
C14	0.0290 (12)	0.0229 (11)	0.0285 (12)	0.0006 (9)	0.0181 (10)	0.0011 (9)
C15	0.0274 (11)	0.0238 (11)	0.0366 (13)	0.0001 (9)	0.0205 (11)	0.0034 (10)
C16	0.0338 (13)	0.0267 (12)	0.0448 (15)	−0.0013 (10)	0.0228 (12)	0.0005 (11)
C17	0.0395 (15)	0.0278 (13)	0.065 (2)	−0.0071 (11)	0.0282 (15)	−0.0017 (13)
C18	0.0493 (17)	0.0341 (15)	0.082 (2)	−0.0043 (13)	0.0429 (18)	0.0143 (15)
C19	0.0468 (16)	0.0465 (16)	0.0550 (18)	0.0070 (13)	0.0364 (15)	0.0192 (14)
C20	0.0347 (13)	0.0319 (13)	0.0423 (15)	0.0055 (11)	0.0258 (12)	0.0072 (11)
C21	0.071 (2)	0.068 (2)	0.0338 (16)	0.0138 (18)	0.0295 (16)	0.0103 (15)
C22	0.0468 (17)	0.071 (2)	0.0466 (18)	0.0152 (16)	0.0306 (15)	0.0058 (16)
C23	0.0296 (14)	0.0359 (15)	0.0547 (18)	−0.0058 (11)	0.0132 (13)	0.0013 (13)
C24	0.0390 (15)	0.0309 (13)	0.0519 (17)	0.0021 (11)	0.0221 (14)	0.0078 (12)

### Geometric parameters (Å, º)

**Table:**

Si1—N1	1.754 (2)	C11—C12	1.388 (4)
Si1—C22	1.846 (3)	C11—H11A	0.9500
Si1—C23	1.854 (3)	C12—C13	1.402 (3)
Si1—C24	1.859 (3)	C12—H12A	0.9500
O1—C20	1.364 (3)	C14—C15	1.491 (3)
O1—C21	1.429 (3)	C15—C16	1.396 (3)
N1—C14	1.385 (3)	C15—C20	1.407 (4)
N1—H1	0.8800	C16—C17	1.390 (4)
C1—C14	1.366 (3)	C16—H16A	0.9500
C1—C13	1.475 (3)	C17—C18	1.378 (5)
C1—C2	1.477 (3)	C17—H17A	0.9500
C2—C3	1.403 (3)	C18—C19	1.380 (5)
C2—C7	1.417 (3)	C18—H18A	0.9500
C3—C4	1.387 (3)	C19—C20	1.392 (4)
C3—H3A	0.9500	C19—H19A	0.9500
C4—C5	1.391 (4)	C21—H21A	0.9800
C4—H4A	0.9500	C21—H21B	0.9800
C5—C6	1.387 (4)	C21—H21C	0.9800
C5—H5A	0.9500	C22—H22A	0.9800
C6—C7	1.391 (3)	C22—H22B	0.9800
C6—H6A	0.9500	C22—H22C	0.9800
C7—C8	1.459 (3)	C23—H23A	0.9800
C8—C9	1.395 (3)	C23—H23B	0.9800
C8—C13	1.411 (3)	C23—H23C	0.9800
C9—C10	1.383 (4)	C24—H24A	0.9800
C9—H9A	0.9500	C24—H24B	0.9800
C10—C11	1.385 (4)	C24—H24C	0.9800
C10—H10A	0.9500		
			
N1—Si1—C22	109.34 (12)	C12—C13—C1	132.2 (2)
N1—Si1—C23	113.09 (12)	C8—C13—C1	109.0 (2)
C22—Si1—C23	111.28 (16)	C1—C14—N1	121.7 (2)
N1—Si1—C24	103.88 (12)	C1—C14—C15	124.0 (2)
C22—Si1—C24	111.08 (15)	N1—C14—C15	114.3 (2)
C23—Si1—C24	107.95 (13)	C16—C15—C20	118.7 (2)
C20—O1—C21	117.4 (2)	C16—C15—C14	119.0 (2)
C14—N1—Si1	130.40 (16)	C20—C15—C14	122.3 (2)
C14—N1—H1	114.8	C17—C16—C15	121.3 (3)
Si1—N1—H1	114.8	C17—C16—H16A	119.3
C14—C1—C13	127.5 (2)	C15—C16—H16A	119.3
C14—C1—C2	126.5 (2)	C18—C17—C16	118.8 (3)
C13—C1—C2	105.54 (19)	C18—C17—H17A	120.6
C3—C2—C7	118.0 (2)	C16—C17—H17A	120.6
C3—C2—C1	133.2 (2)	C17—C18—C19	121.4 (3)
C7—C2—C1	108.6 (2)	C17—C18—H18A	119.3
C4—C3—C2	119.7 (2)	C19—C18—H18A	119.3
C4—C3—H3A	120.1	C18—C19—C20	120.0 (3)
C2—C3—H3A	120.1	C18—C19—H19A	120.0
C3—C4—C5	121.5 (2)	C20—C19—H19A	120.0
C3—C4—H4A	119.2	O1—C20—C19	123.7 (3)
C5—C4—H4A	119.2	O1—C20—C15	116.6 (2)
C6—C5—C4	120.0 (2)	C19—C20—C15	119.7 (3)
C6—C5—H5A	120.0	O1—C21—H21A	109.5
C4—C5—H5A	120.0	O1—C21—H21B	109.5
C5—C6—C7	118.9 (2)	H21A—C21—H21B	109.5
C5—C6—H6A	120.6	O1—C21—H21C	109.5
C7—C6—H6A	120.6	H21A—C21—H21C	109.5
C6—C7—C2	121.8 (2)	H21B—C21—H21C	109.5
C6—C7—C8	129.7 (2)	Si1—C22—H22A	109.5
C2—C7—C8	108.4 (2)	Si1—C22—H22B	109.5
C9—C8—C13	121.4 (2)	H22A—C22—H22B	109.5
C9—C8—C7	130.3 (2)	Si1—C22—H22C	109.5
C13—C8—C7	108.3 (2)	H22A—C22—H22C	109.5
C10—C9—C8	118.7 (2)	H22B—C22—H22C	109.5
C10—C9—H9A	120.7	Si1—C23—H23A	109.5
C8—C9—H9A	120.7	Si1—C23—H23B	109.5
C9—C10—C11	120.6 (2)	H23A—C23—H23B	109.5
C9—C10—H10A	119.7	Si1—C23—H23C	109.5
C11—C10—H10A	119.7	H23A—C23—H23C	109.5
C10—C11—C12	121.3 (2)	H23B—C23—H23C	109.5
C10—C11—H11A	119.3	Si1—C24—H24A	109.5
C12—C11—H11A	119.3	Si1—C24—H24B	109.5
C11—C12—C13	119.2 (2)	H24A—C24—H24B	109.5
C11—C12—H12A	120.4	Si1—C24—H24C	109.5
C13—C12—H12A	120.4	H24A—C24—H24C	109.5
C12—C13—C8	118.7 (2)	H24B—C24—H24C	109.5
			
C22—Si1—N1—C14	29.8 (3)	C7—C8—C13—C12	177.0 (2)
C23—Si1—N1—C14	−94.8 (2)	C9—C8—C13—C1	179.6 (2)
C24—Si1—N1—C14	148.4 (2)	C7—C8—C13—C1	−0.3 (3)
C14—C1—C2—C3	−5.7 (4)	C14—C1—C13—C12	13.6 (4)
C13—C1—C2—C3	−177.8 (2)	C2—C1—C13—C12	−174.5 (2)
C14—C1—C2—C7	168.4 (2)	C14—C1—C13—C8	−169.6 (2)
C13—C1—C2—C7	−3.6 (2)	C2—C1—C13—C8	2.3 (2)
C7—C2—C3—C4	−0.9 (3)	C13—C1—C14—N1	−167.5 (2)
C1—C2—C3—C4	172.9 (2)	C2—C1—C14—N1	22.2 (4)
C2—C3—C4—C5	0.0 (4)	C13—C1—C14—C15	11.7 (4)
C3—C4—C5—C6	0.6 (4)	C2—C1—C14—C15	−158.7 (2)
C4—C5—C6—C7	−0.3 (4)	Si1—N1—C14—C1	−139.9 (2)
C5—C6—C7—C2	−0.6 (4)	Si1—N1—C14—C15	40.8 (3)
C5—C6—C7—C8	−177.5 (2)	C1—C14—C15—C16	−127.0 (3)
C3—C2—C7—C6	1.2 (3)	N1—C14—C15—C16	52.3 (3)
C1—C2—C7—C6	−174.0 (2)	C1—C14—C15—C20	53.6 (3)
C3—C2—C7—C8	178.7 (2)	N1—C14—C15—C20	−127.2 (2)
C1—C2—C7—C8	3.5 (3)	C20—C15—C16—C17	−0.6 (4)
C6—C7—C8—C9	−4.6 (4)	C14—C15—C16—C17	179.9 (2)
C2—C7—C8—C9	178.2 (2)	C15—C16—C17—C18	0.4 (4)
C6—C7—C8—C13	175.2 (2)	C16—C17—C18—C19	−0.2 (5)
C2—C7—C8—C13	−2.0 (3)	C17—C18—C19—C20	0.1 (5)
C13—C8—C9—C10	1.4 (4)	C21—O1—C20—C19	13.5 (4)
C7—C8—C9—C10	−178.8 (2)	C21—O1—C20—C15	−167.3 (2)
C8—C9—C10—C11	0.5 (4)	C18—C19—C20—O1	178.9 (3)
C9—C10—C11—C12	−0.6 (4)	C18—C19—C20—C15	−0.3 (4)
C10—C11—C12—C13	−1.1 (4)	C16—C15—C20—O1	−178.7 (2)
C11—C12—C13—C8	2.9 (4)	C14—C15—C20—O1	0.8 (3)
C11—C12—C13—C1	179.5 (2)	C16—C15—C20—C19	0.6 (4)
C9—C8—C13—C12	−3.1 (3)	C14—C15—C20—C19	−180.0 (2)

### Hydrogen-bond geometry (Å, º)

Cg1, Cg2 and Cg4 are the centroids of the C1,C2,C7,C8,C13, C2–C7 and C15–C20 rings, respectively.

**Table:**

*D*—H···*A*	*D*—H	H···*A*	*D*···*A*	*D*—H···*A*
N1—H1···*Cg*1^i^	0.88	2.69	3.347 (3)	133
C12—H12*A*···*Cg*4	0.95	2.99	3.750 (4)	138
C16—H16*A*···*Cg*2^i^	0.95	2.65	3.470 (3)	145
C21—H21*C*···*Cg*2^ii^	0.98	2.94	3.736 (4)	139
C24—H24*A*···*Cg*3^i^	0.98	2.99	3.923 (4)	158

Symmetry codes: (i) −*x*+2, −*y*+1, −*z*+1; (ii) −*x*+2, *y*−1/2, −*z*+3/2.
